# “Think positive and don’t die alone” - Foreign-born, South Asian older adults’ perceptions on healthy aging

**DOI:** 10.1080/17482631.2023.2253576

**Published:** 2023-09-10

**Authors:** Diya Chowdhury, Paul Stolee, Joanie Sims-Gould, Catherine Tong

**Affiliations:** a School of Public Health Sciences, University of Waterloo, Waterloo, Canada; b Department of Family Practice, University of British Columbia, Vancouver, Canada

**Keywords:** Culture, immigrant, qualitative, successful ageing, ethnogerontology, well-being

## Abstract

South Asians are the largest and fastest-growing racialized group in Canada, yet there are limited data on various aspects of health and well-being within this population. This includes the South Asian older adults’ ethnoculturally informed perceptions of ageing. The study aimed to understand how social and cultural forces impact the meaning assigned to healthy ageing amongst older South Asians in Canada. We recruited with purposeful and snowball sampling strategies in Southern Ontario. We conducted in-depth focus group and individual interviews (*n* = 19) in five South Asian languages, employing a multilingual and cross-cultural qualitative approach. In our analysis, we identified three central themes: (a) taking care of body (b) taking care of mind and heart and (c) healthy ageing through the integration of mind and body. Our study demonstrates that older immigrants are a diverse and heterogeneous population and that their conception of healthy ageing is strongly influenced by their country of origin. This study also demonstrates how racialized foreign-born older adults might provide distinctive perspectives on the ageing process and on social theories of ageing due to their simultaneous immersion in and belonging to global majority and global minority cultures. This research also adds to the limited body of literature on the theories of ageing, despite migration trends, still has a white-centric lens.

## Introduction

Like other nations in the Global North, Canada is experiencing two notable changes in the demographic landscape: increased immigration and a growing population of older adults (Warnes et al., 2004, Zubair & Norris, 2015). Among the older adult population are growing numbers of immigrants who are racialized (Statistics Canada, 2021). This warrants further exploration of racialized foreign-born older adults’ heterogenous nature of experiencing old age (Reich et al., 2020, Vincent et al.,^2006^). Currently, the experiences of foreign-born older adults are not well-understood, on account of both theoretically- and empirically-limited work (Torres 2020, Torres 2022). South Asians are the largest and fastest-growing racialized group in Canada, yet the culturally unique health needs of this population are not comprehensively studied (see reviews from Koehn et al., 2013, Lin, 2021, Quay et al., 2017, Wang et al., 2019). To our knowledge there is no published data, empirical or theoretical, on the culturally informed perceptions of ageing amongst South Asian older Canadians (or any racialized older Canadians). This study aims to understand how social and ethnocultural influences impact the meaning assigned to healthy ageing amongst older South Asians in Canada, adding the voices of racialized older adults to the discussions on healthy ageing.

## Background

### Defining and differentiating successful and healthy ageing

In an era of migration, and global ageing, it is important to re-examine the definitions of “healthy ageing”, given their implications for health and social care delivery and planning (Behr et al., 2023, Lu et al., 2018, Rudnicka et al., 2020, Torres & Lawrence, 2012). Similar to the companion concept of “successful ageing” (Cosco et al., 2014, Rowe & Kahn, 1998, Rubinstein & de Medeiros, 2015), conceptualizing and operationalizing healthy ageing has long been the subject of vigorous inquiry and debate in gerontology (Abud et al., 2022, Bousquet et al., 2015, McLaughlin et al., 2012). Because both health and ageing are multi-dimensional concepts, capturing the depth of the meanings associated with “healthy ageing” within one universal definition is an elusive task. Various gerontological paradigms and studies have introduced successive terminologies to define the process of ageing, including successful ageing, active ageing, and healthy ageing, amongst others (Cosco et al., 2014, Depp & Jeste, 2006, Friedman et al., 2019, Martin et al., 2015, Urtamo et al., 2019, Wong, 2018). For instance, the World Health Organization (WHO) defines healthy ageing as “the process of developing and maintaining the functional ability that enables wellbeing in older age” (World Health Organization, 2020), while Friedman et al. (2019) point out the widespread use of the biomedical model for defining healthy ageing, where a key focus is on the absence of disease. Conversely, the psychosocial lens on healthy ageing highlights general life satisfaction, social functioning, and psychological resources as key components (Friedman et al., 2015, Wong, 2018).

The term healthy ageing is often used synonymously, and critics would argue incorrectly, with successful ageing (Torres, 2015, Wong, 2018). Historically, several definitions of successful ageing have been identified, which include concepts such social and psychological well-being, adapting personal value to face challenges related to older age, as well as definitions that focus on goal achievement and productivity (Gibson, 1995, Clark & Andersson, 1967, Baltes & Baltes, 1990). However, the best known, and arguably the most applied, model to this day is Rowe and Kahn’s (1997) model of successful ageing (Stowe & Cooney, 2015). This model of successful ageing posits the absence of disease and disability as well as an emphasis on individual well-being and personal satisfaction (Rowe & Kahn, 1997). Despite being the predominantly accepted definition of healthy ageing, successful ageing has been the subject of much criticism. For instance, McLaughlin et al. (2010) showed that only 11.9% older adults were “successfully” ageing according to the definition of successful ageing proposed by Rowe and Kahn’s (1997) conceptualization (McLaughlin et al., 2010, Meng & D’Arcy, 2014). Furthermore, the premise of avoiding disease altogether makes this framework of successful ageing unrealistic for most older adults (Friedman et al., 2015, Stowe & Cooney, 2015). On the other hand, healthy ageing, which is a related concept, encapsulates preservation of physical and cognitive capacity, but does not necessitate the avoidance of disease (McLaughlin et al., 2012, Rowe & Kahn, 1997, Wong, 2018). In effect, Wong (2018) differentiates between successful and healthy ageing by stating that healthy ageing is a concept that is more applicable to the current population of older adults since it is more inclusive than successful ageing. Therefore, for the research presented here, we have opted to use the term “healthy ageing” instead of successful ageing, while drawing on the World Health Organization’s World Health Organization (2020) definition of healthy ageing and being mindful of the fact that healthy ageing extends beyond function and the absence of disease (Friedman et al., 2015).

### Culture and ethnicity

The terms culture and ethnicity are often used interchangeably (Torres, 2015), with both terms often employed as euphemisms for race (Bell, 2015). However, with an increase in international migration and growing communities of diverse older adults, such concepts merit attention as they are frequently conflated to mark distinctions amongst social groups. Several authors have discussed the concepts of culture and ethnicity in gerontology (e.g., Castles, 2000, Cornell & Hartmann, 2006, Jenkins, 1997), therefore, an in-depth examination of all the current and historic definitions is beyond the scope of this paper. Renfrew (1996) points out that a common approach to defining ethnicity focuses on genetic, linguistic, and geographical variables, which often deems ethnic identity as bounded and monolithic despite being socially contingent (Smith & Wilkinson, 2007). Conversely, although the most used definition of ethnicity revolves around a mixture of physical, behavioural, and cultural attributes (Pfeffer, 1998), Jenkins (1997) offers a nuanced definition of ethnicity in differentiating it from culture, i.e., ethnicity is a product of culture that involves shared meaning through both social interaction and self-identification. Torres (2015) furthers this notion by describing ethnicity as a background variable to which a person “belongs” and culture as a variable that adds meaning to the “belonging”. Therefore, similar to culture, ethnic identity can be a subjective social marker that is determined situationally (Torres, 2015, Culley, 2006). The relationship between culture and ethnicity can be summarized by Nagel’s shopping cart analogy: “ethnic boundary construction determines the shape of the shopping cart (size, number of wheels), and “ethnic culture” is composed of the things we put into the cart such as food, music, dress, religion, beliefs, symbols, and customs. Culture is not a shopping cart that comes to us already loaded with a set of goods; rather, we construct culture by picking and choosing items from the shelves of the past and the present (Nagel, 1994). Cultures change, “they are borrowed, blended, rediscovered and reinterpreted” (Nagel, 1994, p. 162). Therefore, particularly for older immigrants, both their countries of origin and their country of residence impact the cultural markers they possess, giving rise to a resultant hybridized cultural identity that is complex, ever changing, and fluid, as opposed to being an immutable characteristic (Ford & Harawa, 2010, Malik, 1996, May, 1999).

Culture is dynamic and multidimensional (Asad & Kay, 2015, Raeff et al., 2020, Sue, 2001). Consequently, the term is viewed through several lenses, often dictated by the respective disciplines of those involved (Cai, 2016, Truong et al., 2014). Several definitions of culture exist in the literature, including defining it as an integrated pattern of human behaviour, a dynamic accumulation of socially and geographically-derived human experience, and the manifestation of distinct social, economic, and political contexts (Cross, 1989, Raeff et al., 2020, Truong et al., 2014). For the purposes of our study, we employ Torres’s (2015) definition of culture, which is specific to the field of ethno-gerontology. In this definition, culture is the dynamic interplay between race, ethnicity, and human experience, and provides a sense of belonging to one’s ethnic group (Torres, 2015). What healthy ageing means to older adults may thus vary depending on their lived experiences, personal circumstances, and social backgrounds. Therefore, just as anthropologists understand culture as open, heterogenous, and changing, understanding healthy ageing can also be seen as malleable and fluid (Perkinson & Solimeo, 2014).

### The role of culture in shaping the beliefs and behaviours of older adults

Torres (1999) and Fung (2013) suggests that since an individual’s cultural background influences their way of life, their value and belief system, as well as their shared process of meaning-making, including how one approaches and interprets their life, inadvertently impacts how individuals construe ageing-related constructs, such as healthy ageing. Specific to how migrating to a new country impacts healthy ageing, Sampaio (2021) suggests the importance of considering local and transnational settings within which identities related to ageing are formed, enacted, and restructured. Studying the influences of translational mobility can enable a broader understanding of how ageing identities are given meaning and how hybrid cultures of the country of origin and the newer country of migration impacts the experience and conceptualization of healthy ageing (Sampaio, 2021).

However, most of the recent definitions of health ageing lack a consideration of culture, perhaps owing to the emphasis on the biomedical model of health (Abud et al., 2022, Cosco et al., 2014, Depp & Jeste, 2006, Friedman et al., 2019, Martin et al., 2015, Urtamo et al., 2019). The theoretical development of healthy ageing is largely rooted within an *assumed* white-centric, Eurocentric paradigm, despite growing evidence that the definitions, conceptualizations, and individual internalizations of ageing are influenced by cultural context (Bar-Tur, 2021). Although everyone has “culture”, this body of research tend to equate culture with “otherness” (Torres, 2022). Decades of research has clearly demonstrated that the understanding of health, ageing, and well-being are influenced by larger sociodemographic factors (e.g., gender, age, race, ethnicity, culture, socioeconomic status) as well as by individual lived experiences (Ryff & Singer, 1996, Ryff, 2018). In their examination of psychological well-being, Ryff (2018) noted that the understanding of domains related to positive functioning, including autonomy, purpose in life, and personal growth, vary by cultural context. Other studies that have contextualized well-being based on work or familial environment have also suggested the potential influence of culture on the understanding of such core aspects of living (Bloch-Jorgensen et al., 2018). Sheldon’ et al. (2004) analysis of subjective well-being has suggested that well-being is impacted by culture, including whether a culture is considered more collectivist or individualist. Similarly, Oishi (2000) reported that goals related to independence and self-expression are often deemed less beneficial within collectivist cultures compared to individualist cultures. Specific to older adults, Martin et al. (2015) have noted that the value system that informs the understanding of ageing can differ between cultures of the Global North and the Global South. As such, culture largely impacts the way in which individuals make sense of their world and how they assign meaning to themselves and their surroundings (Hannerz, 1992, Ketay et al., 2009, Torres, 2006b). Intercultural differences in perceptions of ageing are an important consideration when attempting to define healthy ageing, as there is a gap in the literature in understanding how culture (e.g., cultural norms, beliefs, practices, understandings of ageing) may impact the experience of ageing for racialized immigrant older adults. As Torres (1999) states, “culturally-specific knowledge is necessary if we are to discover what lies behind its many existing constructions” (p. 7).

### South asian older adults in canada

The rapid increase in migration and globalization patterns in the developed world has important implications for health systems, healthcare workers, and the health of individuals (Hamilton et al., 2020, Markides & Rote, 2019). South Asians account for 28.4% of the racialized population in Canada and 7.1% of the entire population (Statistics Canada, 2021). Large groups of South Asian immigrants, particularly from countries such as India, Pakistan, Bangladesh, and Sri Lanka, reside in Toronto, the capital of Ontario, and surrounding regions, namely the Greater Toronto Area (Islam et al., 2017). The vast majority of those from South Asian origin are foreign-born (68%) (Chui et al., 2006). Despite South Asians being one of the largest and fastest-growing racialized groups in Canada, the health and wellbeing of this subpopulation is not well understood (Veenstra & Patterson, 2016), with most work focusing on differences and disadvantages in comparison to the reference group (i.e., those who are Canadian-born and non-racialized) (e.g., Anand et al., 2000, Chiu et al., 2010, Gupta et al., 2002, Kim et al., 2013, Liu et al., 2010, Omariba, 2015, Veenstra, 2009, Veenstra & Patterson, 2016). The lack of data on various aspects of health and well-being of this population, including culturally informed perceptions of ageing, mask possible differences in health attitudes, norms, and practices. To support the health and well-being of racialized foreign-born older adults in an equitable and culturally sensitive manner, it is important to first recognize how this population perceives and understands healthy ageing. Similar to the many possible definitions of culture and ethnicity, it is also important to consider terminologies that are used to describe people belonging to specific cultures and ethnicities. Pfeffer (1998) stresses the need to be aware of the meaning that is retained of the words and terms when used for individuals in isolation and in collective contexts. A common critique of using certain terms is that they lack specificity. For instance, the term “Asian” can constitute people from several different countries, differentiated in their ethnocultural background, such as India, Pakistan, China, or Korea to name a few (Pfeffer, 1998). Similarly, the term “white” can also cover a range of people, such as those of Jewish or Irish origin (Pfeffer, 1998, Sheldon and Parker, 1992). Another key criticism of using terminologies to describe specific populations is that it may lead to homogenization of individuals or communities of diverse national, linguistic, religious, and ethno-cultural backgrounds (Rahemtullah, 2010). Regarding the term “South Asian”, Rahemtullah (2010) states that, although the term seemingly unifies the ethno-racial group, not acknowledging the historic and ethnocultural specificities can be problematic. Moreover, Carsignol (2014) criticizes the “South Asian” category for having monolithic and homogenizing connotations that historically has led to maintenance of unequal power relations between immigrant and non-immigrant groups. In the Canadian context, “South Asian” is a term commonly used to encompass people from a large diversity of ethno-cultural backgrounds, including Bangladeshi, Indian, Pakistani, Nepali, and Sri Lankan ancestry (Statistics Canada, 2021). Thus, while several different terminologies are used by authors to describe their participants, it is important to provide an explanation for why a specific term was chosen over others, at the very least, for comparison purposes with other studies (Pfeffer, 1998).

Recognizing intercultural variations in perspectives of ageing becomes crucial when examining the concept of ageing well, as there exists a research gap in understanding the potential influence of culture (such as cultural norms, beliefs, practices, and perceptions) on the ageing journey of older adults from racialized immigrant backgrounds. The purpose of this study is to describe how foreign-born (i.e., immigrant) South Asian older adults in Canada define and conceptualize the notion of “healthy ageing”, thus contributing to the healthy ageing literature which (despite calls for a more culturally responsive lens, e.g., Torres, 1999) has overemphasized a white-centric- lens (Fung, 2013).

## Methods

To understand how foreign-born South Asian older Canadians define healthy ageing, we conducted focus group and in-depth individual interviews in five South Asian languages, employing a strengths-based, multilingual, and cross-cultural qualitative approach (Liamputtong, 2010). The chosen methodology for this study is qualitative description, the objective of which is to provide a comprehensive summary of particular events experienced by individuals or groups of individuals (Kim et al., 2017). A cross-cultural qualitative approach is used to examine and interpret cultural phenomena, behaviours, beliefs, or experiences across diverse cultural contexts (Chan, 2008, Liamputtong, 2010). Researchers aim to comprehend and compare the commonalities and distinctions among various cultural groups, employing qualitative techniques such as interviews, observations, focus groups, and content analyses (Jackson & Niblo, 2003). The primary objective is to gain insights into how culture impacts individuals and societies, leading to a greater understanding of how cultural elements influence attitudes, behaviours, and perceptions (Jackson & Niblo, 2003, Liamputtong, 2010). Identification of existing strengths to create positive, long-lasting change is the main tenet of a strengths-based strategy (Reed et al., 2008, Saleebey, 2006). According to Fast and Chapin (2002), the foundation of a strengths-based approach is based on the innate capacity of people to develop, heal, and learn, as well as on their capacity to recognize wants, the positive aspects of the individual and environment, as well as their sense of autonomy, individuality, and uniqueness.

### Setting, context, and recruitment

We conducted this research in the Southern region of Ontario, Canada, one of the major destinations for all immigrants to Canada and representing a range of diverse South Asian communities (Statistics Canada, 2023). We recognize the term “South Asian” may be homogenizing term that may or may not be reflective of people’s lived experiences and individual identities. As such, we used languages-spoken, rather than country of origin, to recruit participants. While the chosen languages reflected the most spoken languages in South Asia, this approach allowed us to identify culture-sharing groups bound by language, instead of colonially drawn borders (Alvi & Zaidi, 2017). Eligible participants were South Asian older adults (60+ years), able to provide informed consent and able to complete the interview in Hindi, Punjabi, Tamil, Urdu, Bangla, or English. The languages used in the study represent the most commonly spoken languages in the region. Because we are working with immigrant populations, we employed the WHO’s cut-off of age 60 for older adults, which is consistent with how the term “older adult” has been used in other studies with individuals in South Asia (Marmamula et al., 2020; Prasad et al., 2021; WHO, 2021).

Upon ethics approval from the University of Waterloo’s Research Ethics Board (#40977), we recruited participants through several South Asian community organizations, utilizing a combination of email, recruitment posters, and word of mouth within the communities. Several of the initial participants then went on to voluntarily assist with snowball sampling (Sadler et al., 2010), sharing our recruitment materials with friends and family who met the eligibility criteria. Consent forms were sent in advance of the interviews or focus groups, via email. As a token of appreciation for their time, interview participants received an honorarium in the form of a $20 CAD gift card.

## Data collection

Data were collected during the fourth wave of COVID-19 in Canada (May-June, 2021), and consequently all interviews and focus groups were completed either via the telephone or video call, at a time of the participants’ choosing. We actively collaborated with racialized immigrant older adults during all research phases. To support relationship-building prior to the interviews, we participated in 167 hours of online community events (e.g., community yoga and deep-breathing sessions), in place of the in-person community engagement that we had planned prior to the pandemic. Prior to conducting the interviews, this active engagement with participants allowed the research team to establish rapport and trust with the participants. The research team intentionally adopted this collaborative approach to enhance the rigour and quality of the data collection process and to enable reciprocity between participants and researchers to avoid extractive research practices. Participants were asked to select either the individual or focus group interview option; in total we spoke to 19 South Asian older adults (See Table I for participant characteristics), providing us with enough data to answer the research questions sufficiently. As such, our sample size was informed by Malterud and colleagues’ concept of information power (2016), in which sample size is determined during data collection, based on the richness of the data obtained and the availability of existing theory to build on from the current body of literature. Eight participants requested a spousal dyad interview, five completed an individual one-on-one interview, and six participated in two separate focus groups. The same interview guide was used for focus groups, dyad, and one-on-one interviews—the wordings of the questions were adjusted to fit each interview type; focus groups ran longer than the dyad and one-on-one interviews to ensure the same questions were asked. Participants were given pseudonyms to protect anonymity (Table IV links each pseudonym to demographic information, the language of the interview, the type of interview, etc.).

**Table I.** Participant demographic.

Interview and focus group sample (*n*=19)	
**Age**	71.2 mean (min: 60, max: 86)
**Gender**	
Men	10
Women	9
**Country of origin**	
India	5
Bangladesh	6
Sri Lanka	6
Pakistan	2
**Years in Canada**	26.4 mean (min: 2, max: 52)

We developed the interview guide to start with general questions about healthy ageing, followed by specific probes and follow-up questions. For example, if a person mentioned that food or nutrition was important to healthy ageing- a common response, we would then follow with queries such as: what does food mean to you? How does food support healthy ageing? Data was collected by the first author (DC), who speaks English, Hindi, Punjabi, Urdu, and Bangla (she is a native of Bangladesh and formally studied South Asian languages), and a trained research assistant fluent in Tamil and English. Interviews were often completed in a combination of English and one or more South Asian languages, and were audio recorded. Both interviewers completed comprehensive field notes within 24 hours, and regularly debriefed with one another. Qualitative researchers have recognized the challenges inherent in interpreting and translating, particularly in multilingual (i.e., more than two languages) studies (Inhetveen, 2012). Given the complex and multilingual nature of this data collection, each interviewer was also charged with translating to English and producing an English-language transcript for analysis, using the written translation approach outlined by Inhetveen (2012). The research team met regularly throughout data collection to discuss the interview guide, translation queries, and preliminary findings; these team meetings were recorded.

### Analysis and strategies for rigour

Transcripts were cleaned, anonymized, and uploaded into NVivo 12 for analysis, along with field notes and meeting notes. The interviewers and co-authors met on several occasions to develop the coding structure. We utilized an iterative process of proposing codes and then adding new codes as needed, through line-by-line analysis of the data, which was led by the first author (DC). Our iterative coding strategy is further detailed in Table II. Strategies for rigour included: reflexive memoing while coding (Birks et al., 2008); team meetings throughout the data collection and analysis process; team-based analysis with members representing different disciplines and cultural groups, including “insiders” and “outsiders” (Morse, 2010); and the careful examination of outliers (Saldaña, 2021). Outliers typically refer to participants and/or themes that are unique and distinct from the bulk of the data set (Saldaña, 2021); in this study there was one notable outlier, a participant with notably high socioeconomic status, whose data largely focused on finances and legacy. We used shared memos and team-based analysis meetings to distill and discuss final central themes and address discrepancies in interpretation of the data.

**Table II.** Inductive, Evolving coding Structure.

Initial Codes	Codes added during further reading of transcripts	Codes less salient, either removed or collapsed; not highlighted in the results	Final, high-level clustering of themes and sub-themes, as presented in the results
	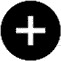	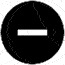	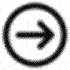
Physical activity Nutrition Mental activity Good genes Healthy habits and lifestyle	Yoga Food as tradition & custom Canadian health care Financial security	Avoiding alcohol and smoking Sleep and rest Good environment (home, community, nature, etc.)	Taking care of body and being • Food: nutrition and sustenance • Physical activity • Healthcare system**s**
Psychological health and outlook socialization Family connections and relationships Spirituality, faith & religion	Adaptation death and dying independence and autonomy collective/community perceptions on ageing	Pets as companions	Taking care of mind and heart • Outlook: positivity, Adaptation, and disposition • Religion and spirituality • Social interaction • Family
	Yoga- mind and body Holistic approach to ageing		Integration of mind and body • Food: sustenance and tradition • Physical activity and spirituality

## Results

At the opening of each interview, most participants consistently commented on the importance of food, fitness, and friends when asked about what is important to their health and well-being. Participants also highlighted that the three core concepts—social interaction, food, and physical activity—are inter-related. For instance, when asked about the importance of one of the core components over the others, participants highlighted how each factor informs the other:

I think social support, physical activity and nutrition are all interdependently important. You can’t have physical exercise alone, without having good healthy eating. And you cannot be doing all these things if you are isolated because that may make you feel down. Divya, female, 71 years old

Our analysis illuminated three central themes related to healthy ageing: (a) *taking care of body (*b) *taking care of mind and heart;* and (c) *healthy ageing through the integration of mind and body*. Table III summarizes the themes and sub-themes and additional details about the participants can be found in Table IV, at the end of this section.

**Table III.** Summary of themes and sub-themes.

Theme	Sub-themes
Taking care of body	Food: nutrition and sustenance
	Physical activity
	Healthcare system
Taking care of mind and heart	Outlook: the importance of positivity, adaptability, and disposition
	Religion and spirituality
	Social interaction
	Family
The integration of mind and body	Food: sustenance and tradition
	Physical activity and spirituality

**Table IV.** Interview/Focus group participant Pseudonyms and demographic information.

Pseudonym	Age	Gender	Country of Origin	Religion/Faith System	Length of time in Canada (Years)	Type of Interview Conducted	Language interview conducted in
Ashish	88	Male	Bangladesh	Hinduism	29	Individual	English
Sanjay	62	Male	Bangladesh	Hinduism	29	Individual	Bengali
Aruna	79	Female	Bangladesh	Hinduism	15	Focus group	Bengali
Prisha	63	Female	Bangladesh	Hinduism	6	Focus group	Bengali
Aparna	76	Female	India	Hinduism	51	Focus group	English
Nivaan	86	Male	India	Hinduism	47	Focus group	English
Zarina	60	Female	Pakistan	Islam	20	Dyad	Urdu
Irfaan	68	Male	Pakistan	Islam	21	Dyad	Urdu
Mahmud	83	Male	Bangladesh	Islam	2	Individual	Bengali
Jay	75	Male	India	Hinduism	52	Individual	English
Divya	71	Female	India	Hinduism	45	Individual	English
Ikshan	77	Male	Sri Lanka	Hinduism	26	Dyad	Tamil
Danvir	78	Male	Sri Lanka	Hinduism	31	Dyad	Tamil
Ashini	73	Female	Sri Lanka	Hinduism	25	Dyad	Tamil
Kanish	72	Male	Sri Lanka	Hinduism	27	Dyad	Tamil
Tarala	78	Female	Sri Lanka	Hinduism	28	Focus group	Tamil
Geeta	82	Female	Sri Lanka	Hinduism	28	Focus group	English
Swapan	64	Male	Bangladesh	Hinduism	9	Dyad	Bengali
Aashi	60	Female	India	Hinduism	10	Dyad	Hindi

### Taking care of body

According to participants, healthy ageing requires the ability to look after the body, through food, physical activity, and adequate access to health care.

#### Food: nutrition and sustenance

Of the three core factors (food, fitness, and friends), the importance of the association between food and healthy ageing was emphasized by most participants. This claim was often accompanied by the notion that food and nutrition were more important than any other factors related to ageing and health:

I would say diet is the most important. If you don’t have healthy body and organs, then you can’t do anything at all. If I eat well, then I can exercise better, and then I have more energy to socialize better as well … so, it’s the most basic need. Mahmud, male, 83 years old

Participants also described, with a particular emphasis on older bodies, that eating high quality, “age appropriate” food, at appropriate times, was essential.

With food … not eating a lot and eating on time are important. We need to eat early. That is something that you can digest quickly. People who are older need to eat early in the morning, in the afternoon, and night … if they eat like that, they will feel healthy. Ashini, female, 73 years old

Many participants discussed the fluctuating nature of the relationship between age and food. This idea of how people’s relationship with food changes based on their age and life circumstances was further highlighted by Prisha when discussing her status as a widow:

This [food restrictions for widows] is a custom. After my father died, my mother only ate one meal a day because her husband was no more. She couldn’t eat any fish, meat, or onions. She would eat only vegetarian dishes. When my husband passed away, I used to do the same thing back home, and it made me weak physically, since I had no protein. But now that I’m in Canada, my children told me to eat more and not restrict myself the same way, so now I eat everything. I don’t have the same level of restrictions here in Canada as my mother did back home. [in Bangladesh] Prisha, female, 63 years old

Prisha’s quote exemplifies how food-related lifestyle choices and habits change for some participants not only in response to age, but also social circumstances and life events. Prisha further explained that moving away from her country of origin led her to ease off on her food restrictions despite widowhood, which also improved the quality of her diet. It is worth noting that Prisha’s decision to ease her food restrictions in Canada was also influenced by her children, underscoring the significant role children can play in their parents’ dietary choices. Their encouragement played a part in her decision to make changes to her diet, illustrating the impact of familial dynamics on food habits in a new environment. Similarly, other participants have also noted that they ate better after moving to Canada; this was attributed to both a higher food quality and a lower need to follow social conventions, such as food restrictions for widows, after migrating.

#### Physical activity

All participants categorized physical activity as an important component of healthy ageing. Some participants also expressed the view that the primary marker for “being healthy” is having the ability to be physically active. Furthermore, many participants indicated that the need to be physically active is higher for older adults compared to other age groups because physical fitness allows them to be independent for longer. When asked about how physical activity may impact healthy ageing, Nivaan explained:

Age is no barrier if you are physically fit, you can move about you’re not dependent on anybody for all activities of daily living, like eating, grooming, taking a bath, or taking a walk … To me, especially in Canada, being physically active is very important so that I will still be able to drive myself. Because you don’t have chauffeurs here like in India. Nivaan, male, 86 years old

Some participants did not equate physical activity with exercise. Instead, they considered it to be able to work and contribute financially to themselves and their family, as stated by Swapan:

If you are not physically active, then you cannot earn … you have to depend on your family for money. But if I can work then I can make money and use it. And even give it to my children if they need it. This can impact the family dynamic too - we are immigrants … I will be a burden if I come [immigrate] here and keep asking them for money. Swapan, male, 64 years old

While physical activity was cited as an important component of healthy ageing, interviews also showed how this facet of healthy ageing is closely related to the immigrant experience and concepts of independence, autonomy, and self-sufficiency.

## Healthcare *system*

The previous sub-themes portrayed participants’ actions in self-care (i.e., eating healthy and exercising), however, participants also emphasized the healthcare system as an additional resource to support them when medical assistance is needed to provide care for their health. The vast majority of older adults stated that a good quality healthcare system is a key component of an environment that supports healthy ageing. Participants frequently used a comparative lens to contrast the Canadian healthcare system to the one they had in their country of origin. Although they recognized a number of limitations (e.g., wait times, language issues, lack of culturally competent long-term care options), participants largely expressed positive views and perceptions towards the Canadian healthcare system. Prisha, who is a newcomer to Canada, expressed her views on why she preferred the Canadian healthcare system over the one in her country of origin:

Even if you don’t have people with you [in Canada], you can call 911 and medical services will come to get you. But back home you don’t have the luxury of that, even if you have money. You need family for that. Or here, you can go to long-term care homes if you can’t live with family. But back home you don’t have long term care homes that are as good as the ones here. Prisha, female, 63 years old

Prisha’s quote is an example of the positive assessments that many participants voiced. Her quote also demonstrates that the shortcomings of the healthcare system in their country of origin may lead immigrants to positively view the healthcare system in Canada. When asked about what impacts his quality of life, Kanish noted:

What I feel is the medical care that we get is one of the main reasons why our people are living longer here [in Canada], than in our own country [Sri Lanka]. If we don’t go, the doctors keep on phoning in and asking us to come for a follow up … So, it is the ongoing support and healthcare that we get here is enabling our people to live longer. Kanish, male, 72 years old

### Taking care of mind and heart

In their discussions on healthy ageing, participants emphasized the importance of nourishing spiritual and emotional well-being, taking care of the mind and heart, and noted various avenues through which this could be accomplished.

#### Outlook: the importance of positivity, adaptability, and disposition

The need to be resilient, adaptive and hone a positive mindset as one ages was frequently expressed by participants. Participants stressed that a positive outlook helps cope better with daily affairs of life and is particularly important for older adults since it allows them to adjust to changes, both mental and physical, that occur with age. Ashish noted how a positive outlook greatly helps with overcoming challenges:

An ability to bounce back … comes with an open mind and positive attitude. If you fall, and you break your leg, you definitely want to believe that you’ll be able to walk one day … if you give up hope then you will never get better … Being resilient is difficult, but there is no way you can keep on brooding. The worst has come but you have to live with your fate with a positive attitude. Ashish, male, 88 years old

Just as Ashish mentions how being resilient can be difficult, many participants have also noted that maintaining a positive attitude can be particularly challenging when one ages. As a solution, many participants voiced that the way to adapt with age, keep a positive outlook, and be resilient was through the removal of expectations. Sanjay explains this from his perspective,

It [staying positive] can become very difficult sometimes when you are old, because when you are young, you could just brush off so many things in life because there is still a long journey in front of you. But as people grow old, they’re coming towards the end of the journey … so, there is the tendency of overthinking or becoming emotional. For example, when I was young … I took care of my parents … And now I look back and see how my kids are taking care of me. If there is any gap in the expectation, then it can be hard … then it definitely contributes to feeling bad. So, keeping positivity intact is very, very difficult sometimes … but it is important. - Sanjay, male, 62 years old

Sanjay’s quote highlights that an individual’s mentality and outlook can fluctuate over their lifespan. Similarly, other participants noted that when faced with an obstacle, it is difficult but important to adjust and evolve accordingly, in order to age well and stay well.

#### Religion and spirituality

Most interview and focus group participants deemed religion an important component of healthy ageing. Tarala notes her perspective on the purpose of religion for ageing well,

Religion is the thing that gives you support to go through all your problems. And as you get older, you take solace in the fact that these problems are given by God … we have to go through these and you pray to God, and you think he will give you an answer. So, you leave it to God every day when you get up … in the morning, you say your prayer and leave everything to God and that takes the weight off your head. Even if you fall ill, you take it as God’s will. Tarala, female, 78 years

In this quote, Tarala explains that religion promotes her mental well-being not only by allowing her to gain comfort during times of distress, but by also holding religion as an explanation for the distress.

Participants, regardless of their faith system, commonly expressed the belief that one’s soul or spirit persists after biological death. In this regard, participants brought up the notion that as they age and near the end of their lives, the inclination towards religion increases as a means to understand what may happen to them in the afterlife. Aruna took this notion one step further to explain that her decision to follow religion comes down to the possibility of an afterlife:

We think of the afterlife as we grow older. You know, what will happen to me after I die? I think of it as a gamble. If praying might grant me a good afterlife, then I’ll just pray … just to be safe. But perhaps it’s for nothing. Who knows? Aruna, female, 79 years old

Many participants highlighted that religion is a prominent source of positivity that impacts their mental and emotional well-being. For instance, Zarina explained that death is not a negative phenomenon because her religion teaches her so:

Aging and death is a positive thing as long as you follow what God says. But yes, everyone will die. That’s a fact. You have no control over that, but what happens to you after that, is dependent on your actions. … So, we don’t see death and aging as a negative thing, but rather as the beginning of a more permanent life. Zarina, female, 60 years old

Although most participants voiced similar ideas and the belief that death is not the end, some other participants disagreed. Of the participants who asserted that religion is of little or no importance for healthy ageing, most made a distinction between religion and spirituality:

Religion is an institution - it dictates. It is manmade and ritualistic. And you are told to believe in one particular type of God. You cannot go beyond one group and community. But spirituality has no limits. Sanjay, male, 62 years old

The quotes above highlight the view that, although typically used as interchangeable terms, religion and spirituality may represent different ideas. Participants shared the view that religion is rooted in collective thinking at the level of community and organization, while spirituality operates on a more personalized level. However, despite the differences or similarities between the two concepts, participants agreed that both play a critical role in healthy ageing, as a means of providing mental clarity as well as emotional and social support.

#### Social interaction

Participants largely identified social interaction as a means of promoting and fostering positive mental health and well-being. It was also emphasized that social interaction becomes increasingly important as one ages. For instance, Mahmud highlighted that social interaction was a way to lessen the state of isolation that increases with age:

Socialization is so important. You need people to talk to and interact with, or else you will become very lonely. You can have everything right – food, exercise, or money – but if you don’t have anyone to speak with or spend time with, then everything feels like it doesn’t matter. Especially as you age … you want to socialize more. Mahmud, male, 83 years old

Several participants have stated that social interaction is particularly challenging for foreign-born older adults because having immigrated, aside from their spouse and children, they lack other familial connections. Commenting on why social interaction for foreign-born older adults can be particularly challenging, Geeta noted:

As you get older, active social life is a problem. Humans are social animals … but isolation is the biggest problem with old people here. Isolation is a big problem for them, and social gathering is very difficult for them. Even your very own children … have no time. And especially for immigrants, it’s a big problem because in our country of origin we don’t have to go out to reach out to others. They come here into your house … your relatives, your children, your neighbors. But here, you don’t know your very next-door neighbor. Geeta, female, 82 years old

Similar to Geeta, Irfaan noted how the frequency of socialization was greater for him in his country of origin:

Back home the culture of socializing would be different. We would be with family a lot more. Here we don’t have that many relatives or family members … we have more friends here. The dynamic of socializing would be different if we were back home and not here [in Canada]. We also meet people less here. Back home we would socialize more. Irfaan, male, 68 years old

#### Family

Most participants spoke of their family, particularly their children and spouse, as the primary source of emotional and social support, both of which were identified as important components of healthy ageing. As noted by Aparna:

You need family for emotional support. No other place to get that but from your families. Aparna, female, 76 years old

Apart from emotional and social support, the notion of dependence and reliance was also frequently associated with family, particularly with participants’ children. Aruna, who has been in Canada for 15 years, noted:

I can’t do anything without my family. I don’t even know my address or where the next street is. So, I am completely dependent, because I don’t have an option but to rely on them. Aruna, female, 79 years old

This notion of reliance on family for practical and emotional support was particularly emphasized by newer immigrants. For example, when probed about why the importance of family is greater in Canada, Aashi noted:

Family is more important here than back home because family is all we have here. Back home we would have relatives and friends who could provide more support to us. But since we came to Canada, we have only one source for that. Aashi, female, 60 years old

### The integration of mind and body

Participants discussed healthy ageing as having multiple dimensions. Facets of healthy ageing work together to add meaning to the lives of the participants. Some notable examples of these overlaps were related to the meaning of food and the meaning of exercise.

#### Food: sustenance and tradition

Participants regarded food not only as nutrition, but also as a source of tradition, culture, and connection. Tradition was highlighted by participants as a key form of community-bonding and the primary way of upholding the collective values of their sociocultural background. Therefore, tradition was an additive component to their mental health and emotional well-being. For instance, Prisha explained how food is a form of tradition that allowed her to gain an increased sense of belonging:

Food is a form of tradition. When we eat with my family, my son, and my daughter-in-law, eating is a lot more meaningful. So, those times, when we eat together, I don’t feel like I am older than them or that I am part of a different age group. I feel like one of them. I think that’s important … just eating good nutritious food doesn’t just cut it. It’s important to have that sincerity and togetherness when we eat. Prisha, female, 63 years old

Many participants voiced how food is largely the focal point of much of South Asian celebrations, religious events, and cultural ceremonies:

In terms of tradition, food is a big part of our culture. We have food in all big events, and we like to eat a lot too. Irfaan, male, 68 years old

Food is for sure a tradition. It’s also a matter of eating healthy ethnic food. Food is also a big part of celebrations, gatherings, and invitations that we receive. Zarina, female, 60 years old

The quotes above highlight participants’ view of how the impact and role of food goes beyond bodily functionality, as it impacts mental, emotional and social well-being as well. Aashi’s comment, “*This is just our culture*”, stresses the complex relationship that participants have with food and how integral food can be to their culture.

#### Physical activity and spirituality

Participants highlighted the integration of the mind and body, particularly when discussing spiritual/religious practices and physical activity. The most frequently cited example of this was the practice of yoga. Participants, primarily those who immigrated from India, commonly spoke of yoga as their primary form of physical activity as well as their way of practicing their spirituality and connecting to a higher power. When asked to elaborate about how yoga helps with healthy ageing, Jay noted,

Yoga is all about the mind and the body and how the two are connected. From a medical- neurological standpoint, yoga has so many benefits … it reduces my stress on so many levels and helps me keep active … But at the same time, yoga and its principles have also has gotten me closer to God and taught me to see the beauty in God’s creations. Jay, male, 75 years old

Jay’s comments illustrate how the domains of mental and physical health can be interconnected. Participants who had similar insights also frequently referred to Hinduism as the underlying basis of yoga. Other participants have also specified that their main source of mental clarity, cognitive well-being, stress management, spirituality, as well as exercise, was through the practice of yoga.

Another example of the integration between physical activity and religion/spirituality was through the practice of Islamic prayers, termed “namaaz”. Participants, not only exclusive to the Islamic faith, have emphasized that namaaz, which is a process that requires extensive body movements, including standing, sitting, and bending in a repetitive motion, is a way to cater not only to their mental well-being, but also their physical health. When asked about the role of exercise for healthy ageing, Mahmud noted:

I pray [namaaz] five times a day. And I think that contributes to healthy aging. This is because not only am I getting exercise while praying namaaz, but I also get peace of mind because … I devote myself to God- it brings me peace. Reminds me of a higher purpose of life … and helps me mentally. Mahmud, male, 83 years old

Similar to the practice of yoga, Mahmud’s explanation of namaaz also portrays how mind and body can integrate to form a cohesive understanding of the participants’ view of healthy ageing. This also highlights how integral cultural influences are to activities that participants choose to do to keep themselves mentally and physically healthy.

## Discussion

The present study explored how foreign-born South Asian older adults in Canada perceive and define the concept of healthy ageing. Three primary interrelated dimensions of healthy ageing were highlighted by the participants: (A) taking care of body; (B) taking care of mind and heart; and (C) the integration of mind and body. The multi-faceted views of the participants within each dimension illuminated the influence of culture that remains central to, and informs, their conceptualization of healthy ageing. This finding is consistent with previous research on racialized foreign-born older adults. For instance, in their study with foreign-born Chinese and South Asian older adults, Tong’ et al. (2020) noted that culture is a prominent driver of health behaviours. Similarly, Napier et al. (2014) states that, “ideas about health are … cultural” (p. 17), pointing towards how perspectives on physiological and psychosocial wellbeing differ substantially across and within cultures and societies.

Participants believed that healthy ageing extends beyond simply the absence of disease and longevity. The quality of life, including the quality of food, social connection, and the environment, was highlighted as a key determinant of healthy ageing. Participants also heavily emphasized the importance of attitude, mindset, and outlook that impacts all facets of the process and experience of ageing. This finding is distinct from the biomedical model of ageing that still predominates in the understanding and practise of healthy ageing in the Global North. Rowe and Kahn’s (1997) model, which despite considerable academic criticism is widely cited and influential, highlights three components of successful ageing: avoidance of disease and disability, the maintenance of high physical and cognitive function, and being engaged socially and productively. In contrast, our findings indicate that healthy ageing consists of broader and more holistic domains. Although participants did voice the importance of avoiding disease and disability and having the ability to function well in order to age well, it was not limited to those areas. For instance, participants, to a large extent, highlighted the importance of familial connection and social interaction as well as religion and spirituality as core components of healthy ageing. As such, the findings of this study are consistent with the life course perspective, which posits the importance of the larger historical, social, and cultural context of ageing (Hanson et al., 2016, Kuh et al., 2014). More specifically, the life course approach considers how sociocultural forces and individual agency as well as lived experiences interact to shape the outcomes and experiences of ageing (Dannefer, 2012, Kuh et al., 2014). An example of this in our study is provided by Prisha, a widow who, after moving to Canada, altered her diet due to the greater ease in social restrictions within her new social environment.

Cartesian dualism, a concept credited to 17^th^ century philosopher Rene Descartes, largely dominates the white-centric biomedical understanding of health and ageing, suggesting that physical health (body) and mental health (mind) are separate and distinct (Descartes et al., 1952, Thibaut, 2018). This view of mind and body dichotomy was not shared by many participants in this study. Instead, participants voiced that not only do mind and body inform each another, but that they are, in many instances, integrated. The participants brought up two particularly prominent examples of this: (a) viewing food as sustenance as well as a fundamental source of tradition and social connection, and (b) perceiving physical activity as exercise as well as a means of practicing one’s spirituality and/or religion. Participants explained that although food and physical activity are important for the body, they are just as important for the mind. Although there are several research studies that associate physical activity with mental well-being, such studies focus on the indirect causal pathway that results from exercise, attributed to release of certain hormones and chemical changes that may work to prevent mental disorders (Bize et al., 2007, Peluso et al., 2005). Similarly with food, there is substantial evidence that supports how consumption of healthy and nutritious food may prevent disease burden, such as with obesity and heart disease, which may then consequently promote mental well-being (Owen & Corfe, 2017, van der Pols, 2018). However, this is distinct from the findings of our study because participants emphasized that, as opposed to being a product of eating well and exercising, the very act of doing so, in and of itself, contributes to their mental, spiritual, and social well-being. Participants have attributed this understanding of health to their cultural and social upbringing. In support, Mehta (2011) states that the dichotomous view of mind and body persists because our understanding of health is rooted within a dualistic biomedical culture. However, as exemplified by the participants of this study, it is important to acknowledge the dynamic nature of human beings that results from different cultures and environments and endorse a holistic view of healthy ageing (Ernst, 2007).

Our findings highlighted the significance of family and the predominance of a family-oriented approach to ageing and well-being for our participants. While some participants, particularly those who were newcomers, emphasized their reliance on family for needs such as their emotional well-being and healthcare decision-making, several participants also stressed the importance of fostering interdependence. Prior studies with racialized immigrant older adults that report similar findings on the role of family point towards the understanding that collectivist societies, such as South Asian communities, have a heightened level of importance accorded to family (Conkova et al., 2020, Beyene et al., 2002, Nieboer et al., 2021). For instance, a key finding by Choudhry and colleagues’ (2002) study on South Asian women in Canada reported that participants placed the needs of their family before their own needs—a thought similarly expressed by participants of our study. Another study by Beyene et al. (2002) showed that for Latino older adults in the United States, successful ageing was closely related to and influenced by familial relationships and the fulfilment of culturally embedded expectations from family members, particularly their children. Participants of our study added a step further to explain that the conscious removal of such expectations through the adaptation of their mindset and outlook is a way of reducing stress and upholding their mental and emotional well-being as they age. Barbaranelli et al. (2019) reports the importance of outlook in encouraging people to view their own lives in a positive way, while numerous articles have emphasized the value of optimism in fostering health, happiness, and preventing maladjustment (Alessandri et al., 2012; Caprara et al., 2017). Participants also added that countries in the Global North such as Canada tend to focus more on nuclear families, whereas their countries of origin place a much higher emphasis on communal living, community, and extended social networks beyond immediate family members. As such, our findings are consistent with previous studies that found autonomous or independent control to be less valued and filial piety and familial bonding to be regarded as significant in collectivist immigrant communities (Nieboer et al., 2021, Huang & Fiocco, 2020).

Furthermore, participants conveyed that the influence and impact of their families was not limited to the social domain of healthy ageing. A key example of this brought forth by participants represented an intersection between nutrition, family, religion, and gender. Some female participants emphasized widowhood as a prominent identity marker which influenced not only their social dynamics (e.g., being given less importance and less of a voice since the death of their husband) but also their eating habits. Certain South Asian communities impose restrictions and external markers upon women to indicate their transition to widowhood (Jacobsen & Myrvold, 2018). Participants of our study highlighted that such impositions that are placed to uphold religious and societal norms have negatively impacted their health and experience of ageing. For instance, following the death of their husbands, participants experienced physical weakness on account of a limited diet. However, some participants also conveyed that after immigrating, their children, who brought them to Canada, talked them into easing off on previously imposed dietary restrictions. This reflects a shift in familial dynamics post immigration where the children start to take precedence over their late husbands. Unlike our findings, a study by Vesnaver et al., (2015) that focused on non-immigrant older women from Caucasian or European descent, found that widowhood resulted in re-adjustment of food behaviours that led to more satisfactory personal food systems. Additionally, contradictory to our findings, previous studies that looked at non-immigrant older women reported that with the removal of food-related obligations as a wife, women had more freedom to choose their own food preferences during widowhood (Quandt et al., 2000, Johnson, 2002). Although studies have looked at the relationship between food and widowhood, to our knowledge, this is the first to discuss this relationship between food and widowhood amongst South Asian-born older adults in Canada, which could explain the disparity in findings.

Finally, the results of our analyses also suggested that immigration largely shaped the participants’ experiences and understanding of healthy ageing. Participants recurrently viewed ageing, health, and well-being through the lens of an immigrant, highlighting several challenges, such as decreased opportunities for social interaction, and benefits, such as less stringent cultural norms, that resulted from migrating to Canada. These findings highlight the large influence that immigration has on health behaviours and perceptions of ageing-related constructs among racialized foreign-born older adults. This also suggests that the process of immigrating to a country with different cultures and social norms can influence the understanding and practice of one’s own culture of origin, which then impacts their health behaviours, perceptions, and overall approach to well-being. For instance, in our study we saw that migrating to Canada led to lesser food restrictions for widows. Moreover, Torres (2001) reported similar findings while studying successful ageing amongst Iranian immigrants, noting that individuals often redefine concept of ageing well after immigration. This is particularly so when individuals immigrate to a culture that is different in fundamental ways from their culture of origin, such as migrating from a collectivist society to one that is individualistic (Torres, 2001), as in the case for our study. This was further evidenced by participants of this study who emphasized the need to adapt and evolve their approach and attitude towards everyday life, including health and ageing, to one that was compatible with norms of the Global North, such as with dietary changes and social compliance. Therefore, not only is culture interwoven within all dimensions of well-being, but the approach to and understanding of healthy ageing is contingent upon the individuals’ culture of origin, the culture that is acquired after immigrating to a new country, and a hybrid culture that is a product of the two (Torres, 1999, Torres, 2006b).

## Implications for future research

Our team has employed an interpretive lens, drawing on our own knowledge of South Asian culture and academic literature to hypothesize some of the ways in which this culture may inform perceptions of ageing amongst immigrants to Canada. Shooshtari et al., (2020) found that there are differences between conceptualizations of healthy ageing among ethnocultural group. Our study expands on these findings by examining what those specific differences may be for the South Asian population, which is one of Canada’s largest ethnocultural group. Finally, the results of our study have strong implications for future research that focus on the meaning associated with food not only as sustenance but also an important component of culture and sense of identity for South Asian communities. We had limited opportunity to speak to widows belonging to South Asian communities. Future studies are needed to further study the interaction of widowhood and ageing in a foreign land.

Before creating resources that can support racialized immigrant older adults, it would be important to first understand how health and well-being is conceptualized by this population. This study can be used as a starting point to inform future development and implementation of interventions to support culturally competent, person-centred care of racialized immigrant older adults. For example, currently, there is limited evidence regarding the nature of patient engagement, and the role that family members play within this interaction, for racialized foreign-born older adults (Culhane-Pera et al., 2010). This study can shed light on how racialized foreign-born older adults conceptualize the importance of family, and in turn, help in understanding the role that family can play in the experience of ageing. This understanding of the family’s significance in the ageing experience could serve as a foundation for understanding how family might influence healthcare experiences and navigation among racialized foreign-born older adults.

## Implications for practice and policy

Our research has practice and policy implications related to the health and well-being of, and services for, racialized immigrant older adults. This research can assist health practitioners, government organizations, and policy makers in understanding aspects of health that are important to older adults from South Asian backgrounds. For example, government programs that are designed to cater to the health of older adults tend to focus primarily on the physical aspects of health (Clarke et al., 2003); our research points to the importance of considering health beyond the physical and initiating programs that consider the mind in tandem with the body. The results of this study can also aid in planning and evaluating culturally-sensitive healthcare services that cater to the specific needs of South Asian older adult communities. Specifically, the findings of this study could assist healthcare providers with training that helps them become more sensitive to the distinct requirements, cultural beliefs, and individual preferences of racialized immigrant communities in the context of healthcare related to ageing. Similarly, understanding how South Asian older adults conceptualize healthy ageing can enable health practitioners to understand their approach to health management, health behaviour, and healthcare decision making, which has important implications for healthcare provision and patient-provider engagement. The findings of this study can be used to advance scholarship in work related to promoting healthy ageing for older adults from diverse ethnic and linguistic backgrounds by informing policy and practice change at local and possibly national levels (Lin, 2021).

## Limitations

Ours was a qualitative study. While a quantitative population survey could have achieved more generalizable findings, we feel our research makes a valuable contribution to understanding perceptions of healthy ageing among an understudied South Asian older adult population.

In North American research on ethnocultural groups, the term “South Asian” has primarily represented those from India (e.g., Koehn et al., 2016, Oliffe et al., 2009, Tong et al., 2020). It is a strength of our work to have also reached older adults from Bangladesh, Pakistan and Sri Lanka; however, we recognize that our sample does not include individuals from Afghanistan, Bhutan, Maldives or Nepal and cannot be generalized to the experiences of all South Asians ageing in Canada. Future research, both of our team and others, should be cautious of the term “South Asian” when only representing a portion of the region in our recruitment and data collection. Also, in our experiences both here and in prior work (e.g., Tong et al., 2019), we note that it can be incredibly challenging for people to articulate notions of culture and cultural norms in interviews.

## Conclusion

Understanding how racialized foreign-born older adults perceive healthy ageing, and how their perceptions may inform their health behaviour and decision-making, is an important yet understudied domain (Montoya-Williams et al., 2020). Our study is one of only a few studies (e.g., Keith et al., 1990, Oishi, 2000, Sampaio, 2021) focusing on how racialized immigrant older adults perceive and conceptualize healthy ageing. Further, our study reinforces the notion that immigrant older adults are a diverse and heterogenous population, and that the culture of their country of origin is heavily infused in their conceptualization of healthy ageing. This study also highlights that being immersed in and belonging to both a global majority and a global minority culture simultaneously, racialized foreign-born older adults can offer unique perspectives on the ageing process and on social theories of ageing. With the increasingly diverse geriatric population in migrant-receiving, Global North nations, it is now more important than ever to recognize these unique perspectives and understand how intersecting statuses of “ethnic-minority” and “immigrant” can impact older adults’ health and well-being.
